# Microbiome Single Cell Atlases Generated with a Commercial Instrument

**DOI:** 10.1002/advs.202409338

**Published:** 2025-06-03

**Authors:** Xiangpeng Li, Linfeng Xu, Benjamin Demaree, Cecilia Noecker, Jordan E. Bisanz, Daniel W. Weisgerber, Cyrus Modavi, Peter J. Turnbaugh, Adam R. Abate

**Affiliations:** ^1^ Department of Chemistry and Biochemistry Florida State University Tallahassee FL 32306 USA; ^2^ Department of Bioengineering and Therapeutic Sciences University of California San Francisco CA 94158 USA; ^3^ Department of Microbiology & Immunology University of California San Francisco CA 94158 USA; ^4^ Biochemistry and Molecular Biology Huck Institutes of the Life Sciences Penn State University University Park PA 16802 USA; ^5^ Chan Zuckerberg Biohub San Francisco CA 94158 USA

**Keywords:** microbiome, microbiome single cell atlases, microfluidics, single cell sequencing

## Abstract

Single‐cell sequencing is useful for resolving complex systems into their composite cell types and computationally mining them for unique features that are masked in pooled sequencing. However, while commercial instruments have made single‐cell analysis widespread for mammalian cells, analogous tools for microbes are limited. Here, EASi‐seq (Easily Accessible Single microbe sequencing) is presented. By adapting the single‐cell workflow of the commercial Mission Bio Tapestri instrument, this method allows for efficient sequencing of individual microbial genomes. EASi‐seq allows tens of thousands of microbes to be sequenced per run and, as it is shown, can generate detailed atlases of human and environmental microbiomes. The ability to capture large genome datasets from thousands of single microbes provides new opportunities in discovering and analyzing species subpopulations. To facilitate this, a companion bioinformatic pipeline is developed that clusters genome by sequence similarity, improving whole genome assembly, strain identification, taxonomic classification, and gene annotation. In addition, the integration of metagenomic contigs with the EASi‐seq datasets is demonstrated to reduce capture bias and increase coverage. EASi‐seq enables high‐quality single‐cell genomic sequencing for microbiome samples using a simple workflow run on a commercially available platform.

## Graphical abstract

Single cell sequencing is valuable for dissecting complex systems and uncovering unique features in individual cell types. However, tools for single cell microbial analysis are limited. Easily Accessible Single microbe sequencing (EASi‐seq), which adapts a commercially available single cell platform for efficient sequencing of single microbial genomes is introduced. EASi‐seq enables sequencing of thousands of microbes per run, generating detailed atlases of microbiomes. EASi‐seq offers accessible high‐quality single cell genomic data for microbiome samples on a commercially available platform.

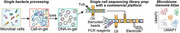

## 1 Introduction

Microbiomes, composed of diverse microorganisms, such as bacteria, archaea, fungi, and virus, are highly complex, often containing thousands of species and billions of cells even in small samples.^[^ ^1^ ^]^ Beyond species diversity, they exhibit significant genetic heterogeneity, driven by differences in core genomes and mobile genetic elements like plasmids and transposons.^[^ ^2^, ^3^, ^4^ ^]^ These mobile elements influence microbial properties and ecosystem dynamics by spreading traits such as antibiotic resistance, transforming benign species into multidrug‐resistant pathogens.^[^ ^5^ ^]^ Detecting these elements and capturing the full genetic diversity of microbial genomes are essential for constructing accurate genome atlases and understanding how genetic heterogeneity impacts microbiome properties. Metagenomic sequencing, which pools and sequences all genetic material,^[^ ^6^, ^7^ ^]^ often requires sophisticated computational algorithms for assembly and sometimes yields disconnected genomic fragments and creates chimeric sequences of different strains or species.^[^ ^8^, ^9^ ^]^

Single‐cell genomic DNA sequencing overcomes these challenges by assigning genetic material to individual cells, capturing incomplete genomes and mobile elements, and linking them to host cells. It also reveals physical associations between cells and phages, providing insights into microbial strain dynamics.

Previous single‐cell sequencing approaches have been based on isolating microbes for single‐cell genome amplification and library preparation using Fluorescence‐activated Cell Sorting (FACS),^[^ ^10^, ^11^, ^12^, ^13^, ^14^, ^15^, ^16^, ^17^, ^18^ ^]^ optical tweezers,^[^ ^19^ ^]^ hydrogel matrix embedding,^[^ ^20^ ^]^ and microfluidics.^[^ ^21^ ^]^ These methods either have limited throughput, allowing just hundreds of genomes to be sequenced, or are highly labor intensive. More recently, barcoding reminiscent to scalable mammalian cell methods have been applied to microbes and achieved the sequencing of similar numbers of cells (thousands of cells per run).^[^ ^22^, ^23^ ^]^ These multi‐step droplet microfluidic approaches utilize robust molecular biology, yielding superb data for most cell types in the sample;^[^ ^22^, ^23^ ^]^ unfortunately, the number of steps and custom‐built instrumentation poses a significant barrier to non‐microfluidic engineers for its application. Another single‐step droplet microfluidic approach enables sequencing multiple gene loci of thousands of single bacteria cells were reported,^[^ ^24^, ^25^ ^]^ however, its application is limited to only a few amplicons. Meanwhile, high‐throughput single bacteria RNA sequencing has been demonstrated using combinatorial indexing,^[^ ^26^, ^27^, ^28^, ^29^ ^]^ commercially available single‐cell platforms,^[^ ^30^, ^31^, ^32^ ^]^ or custom build microfluidics.^[^ ^33^ ^]^ However, these methods have primarily been applied to model organisms. The complex and varied physical properties of diverse microbiomes make it challenging to optimize key steps, such as fixation, permeabilization, and in situ ligation, required for single‐cell transcriptomics. Only recently has single‐cell transcriptome sequencing been successfully applied to complex microbiomes using custom‐built microfluidics.^[^ ^34^ ^]^ This progress highlights both the demand within the microbiology community for high‐throughput single‐microbial sequencing methods and the significant technical barriers that have limited broader adoption. Developing a robust and accessible high‐throughput method would enable more widespread and effective application of single‐cell sequencing across diverse microbiome samples, greatly advancing the field. If such a method could be developed, it would be superior to metagenomic sequencing in most instances and provide access to capabilities currently missed, including generation of complete single‐microbe resolution cell atlases and gene annotation at the strain or single‐cell level.

Here, we present EASi‐seq (Easily Accessible Single‐microbe sequencing), a method for microbial whole‐genome sequencing built on the commercially available Mission Bio Tapestri platform. Originally designed for high‐throughput, targeted sequencing of mammalian cells, the Tapestri platform's two‐step microfluidic workflow provides the flexibility required for microbial applications. Two key innovations enable this adaptation. First, we expanded the range of lysis reagents to ensure unbiased capture of diverse microbes, addressing challenges posed by their robust and varied cell walls. Second, we modified the molecular biology workflow to support whole‐genome sequencing, overcoming the platform's original limitation to targeted sequencing of specific genomic regions. To process the recovered data, we developed a clustering algorithm that groups similar cell types, enabling the reconstruction of accurate consensus genomes from incomplete single‐cell data. EASi‐seq significantly advances microbial genomic research by providing high‐throughput, single‐cell sequencing on a commercially accessible platform.

## 2 Results

### 2.1 EASi‐Seq Workflow for Whole Genome Microbial Sequencing

A reliable platform for sequencing large numbers of environmental microbes must address key challenges, including the diversity of microbial cell walls and membrane properties, which require varied lysis methods.^[^ ^22^, ^35^, ^36^ ^]^ Additionally, genomic and plasmid DNA must be fragmented and adapted for sequencing, and the heterogeneity of microbiomes, often comprising hundreds to thousands of species and strains, demands the sequencing of many individual cells to generate a comprehensive single‐cell atlas. Previous methods relied on custom workflows with 3–5 microfluidic processing steps,^[^ ^22^, ^23^ ^]^ requiring bespoke devices operated by experts, which, while powerful, were inaccessible to many microbiologists. Recent advances in commercial single‐cell platforms (Table S1, Supporting Information), such as Mission Bio's Tapestri, offer more accessible alternatives. Tapestri's ability to perform two sequential droplet microfluidic steps, originally designed for mammalian DNA sequencing,^[^ ^37^ ^]^ provides a foundation for adapting workflows for microbial whole‐genome sequencing. However, replicating earlier microbial workflows directly on Tapestri is impractical due to differences in requirements between microbial and mammalian cells.

**Figure 1:**
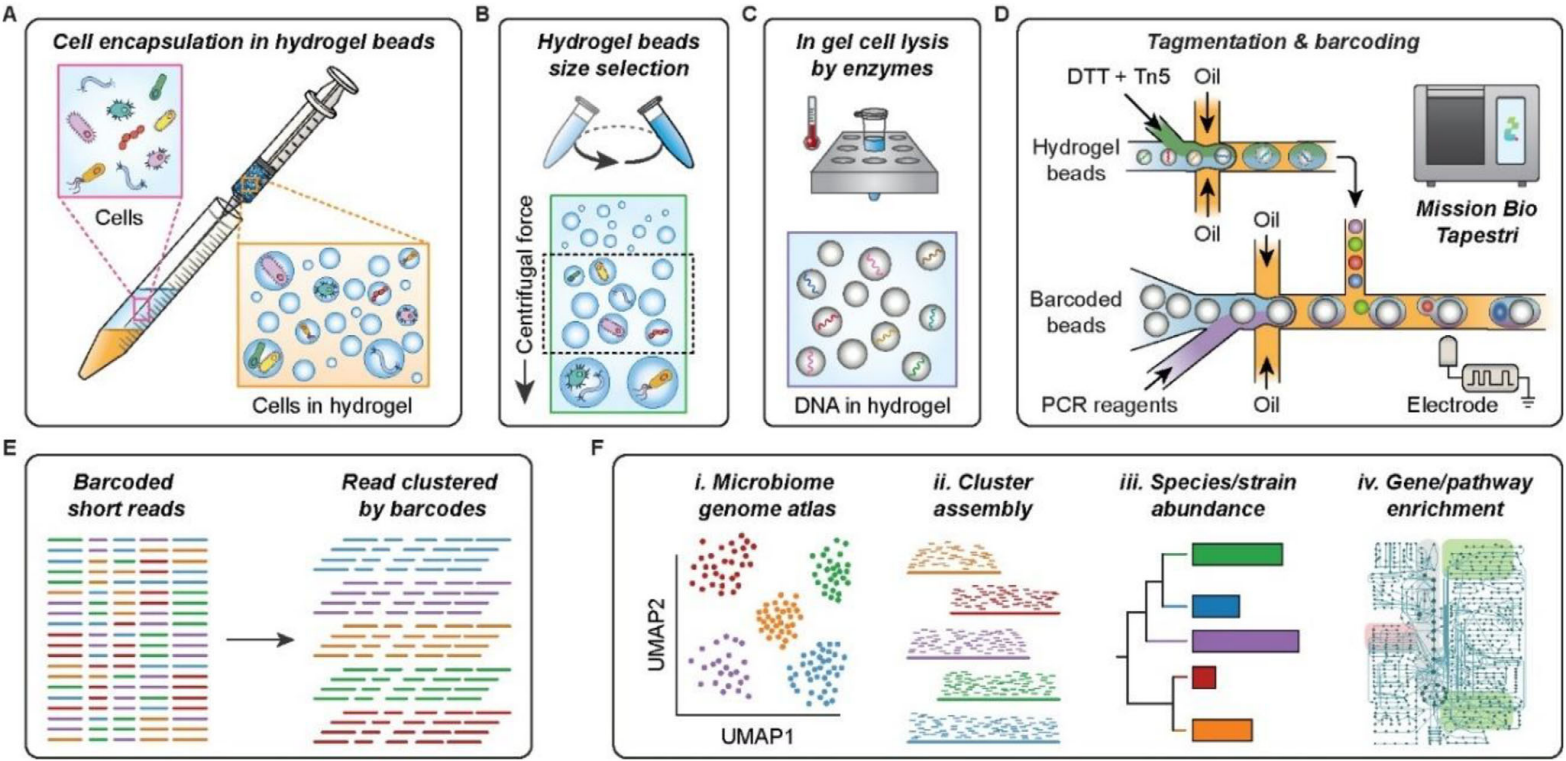
EASi‐seq workflow: Genome purification, microfluidics, and bioinformatics. A) Microbial cells are suspended in a hydrogel precursor solution (acrylamide monomer and N,N’‐bis (acryloyl) cystamine‐BAC crosslinker) and emulsified with fluorinated oil by passing the mixture through a syringe needle. Gelation embeds individual cells within hydrogel beads. B) Hydrogel beads are size‐selected via differential centrifugation. Beads are suspended in a density‐matching buffer (40% sucrose in PBS with 0.1% Tween 20) and centrifuged at 1000 × g for 10 min to pellet oversized beads. The supernatant is centrifuged at 3000 × g for 10 min to pellet the desired bead size (5–30 µm), which is collected as size‐selected hydrogel beads. C) Cells within hydrogel beads are lysed using a two‐step enzyme digestion. A cocktail of four enzymes digests cell walls, followed by Proteinase K treatment to digest proteins. The small pore size of the hydrogel allows diffusion of proteins and smaller molecules while immobilizing long DNA molecules. Washing removes cellular debris, leaving purified genomic DNA trapped within the beads. D) Genomic DNA within hydrogel beads is tagmented in droplets during the first Tapestri microfluidic module (bottom), followed by barcoding PCR in the second module (top), using barcode beads paired with each droplet. E) Sequencing the barcoded amplicons produces single‐cell shotgun reads for thousands of microbial cells. F) EASi‐seq supports high‐throughput microbiome analysis, including genome atlas construction, cluster‐based genome assembly, strain identification, and pathway analysis.

To address this, we developed a novel workflow tailored to Tapestri's two‐step process. Single‐microbe sequencing requires cell lysis, DNA fragmentation, and the addition of single‐cell barcodes. Tapestri's first droplet manipulation is repurposed for DNA tagmentation, while the second enables barcoding. To lyse the cells, prepare the DNA molecules for tagmentation, and preserve both the genome and extrachromosomal DNA, the cells can be encapsulated in hydrogel spheres. This encapsulation enables multi‐step enzyme‐based cell lysis, traps high‐molecular‐weight DNA, and protects it from shear forces during washing.^[^ ^12^, ^15^, ^16^, ^17^, ^22^, ^38^ ^]^ To ensure EASi‐seq's accessibility, we designed a microfluidic‐free hydrogel encapsulation process. Cells are emulsified into acrylamide droplets via shaking or syringe shearing (**Figure** **1**A). Radical polymerization within droplets form polyacryamide hydrogels, preserving DNA integrity and compatibility with PCR and sequencing.^[^ ^38^ ^]^ Differential centrifugation isolates hydrogels of optimal size (5–30 µm) for Tapestri processing (Figure 1B; Figure S1, Supporting Information). Hydrogels are loaded at 2% efficiency into the Tapestri droplets to maximize single‐cell genome recovery. Enzymatic treatments purify genomes while preserving single‐cell integrity.^[^ ^22^ ^]^ Although cell loss is inevitable during hydrogel size selection, but it should not affect species composition in sequencing. Abundant species are more likely to experience cell loss, while less abundant species are less affected, preserving overall statistics. Moreover, microbiome samples typically contain far more cells than EASi‐seq's throughput, minimizing the impact of sample loss.

In Tapestri's first module, hydrogels with purified genomic DNA are liquefied using dithiothreitol (DTT) in the droplets, which cleaves the N,N’‐bis(acryloyl)cystamine (BAC) crosslinker.^[^ ^39^ ^]^ Simultaneously, Tn5 transposase^[^ ^40^ ^]^ is introduced to perform DNA tagmentation, priming fragments for barcoding in the second module. Barcoding is achieved by reinjecting and merging droplets with barcoding PCR reagents, and final sequencing adaptors are added through bulk PCR (Figure 1D; Figure S3, Supporting Information). The resulting material is sequenced and deconvoluted into single cells by barcode (Figure 1E). This workflow generates tens of thousands of single‐cell genomes with 0.01–10% coverage, depending on genome size and sequencing depth. Single‐cell data are clustered into a microbial atlas (Figure 1F, i,ii), and pooled within clusters to create consensus genomes. Integrating metagenomic sequencing can further enhance coverage. Final genus clusters are annotated and analyzed for species or strain abundance and gene or pathway distributions (Figure 1F, iii,iv).

### 2.2 Validation of Single‐Cell Resolution

**Figure 2:**
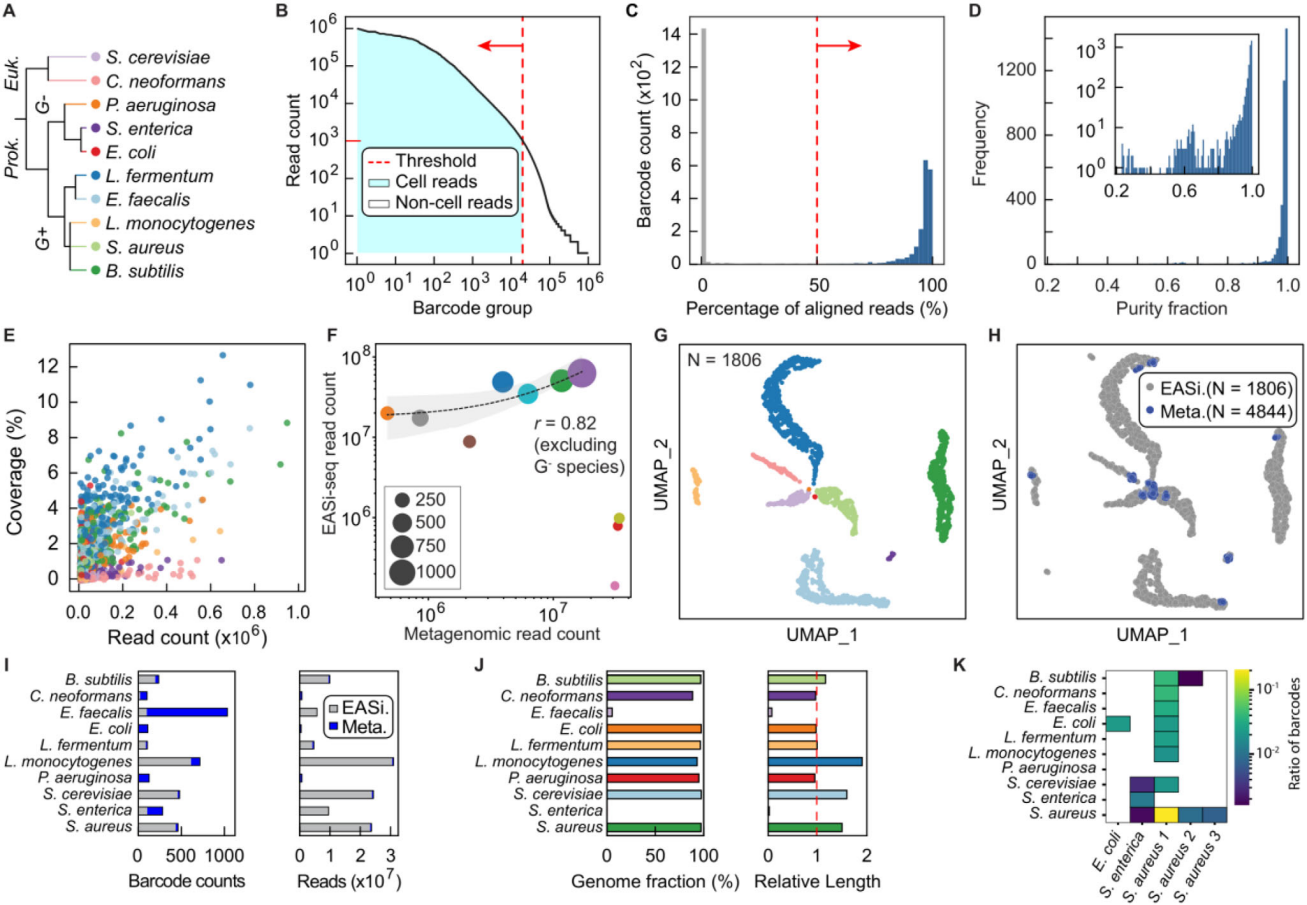
EASi‐seq enables single‐cell resolution. A) The ZymoBIOMICS synthetic microbial community, comprising ten species, was analyzed using EASi‐seq. Species classifications are shown, with assigned colors used in subsequent panels. B) Barcode rank plot of EASi‐seq data, filtered to exclude barcode groups with fewer than 1000 reads. C) Barcode groups were further filtered by alignment rates to reference genomes. Barcode groups with alignment rates below 50% to the combined reference genomes of the 10 species were removed. D) Purity distribution of filtered barcode groups, defined as the percentage of reads mapping to the most represented species within each barcode group. The inset shows the distribution on a logarithmic scale. E) Coverage of barcode groups, color‐coded by species, showing genome representation across the dataset. F) Comparison of metagenomic and single‐cell sequencing data. A scatter plot shows read counts from metagenomic sequencing versus combined EASi‐seq barcode groups. Points are color‐coded by species, with sizes proportional to filtered barcode counts. The regression line (black dotted) and confidence interval (gray shaded) exclude Gram‐negative species. G) UMAP clustering of barcode groups, color‐coded by species, generated using a k‐mer‐based taxonomy classifier (Kraken2). Taxonomic classification was performed at the genus level, and barcodes were filtered by mapped read percentage and taxonomic purity (dominant taxa percentage). The genus abundance vector was used to produce the UMAP, with barcodes annotated by their most abundant genus. Sample size (N) = 1806. H) UMAP clustering shows integration of EASi‐seq (gray, sample size = 1806) and metagenomic (blue, sample size = 4844) data. Contigs from assembled metagenomes were treated as barcodes and processed through the same taxonomy pipeline as EASi‐seq barcodes. I) Barcode and read counts in each UMAP cluster, grouped by batch (EASi‐seq or metagenomic assembly). J) Contig evaluation within UMAP clusters. Reads from all barcodes in each cluster were assembled into contigs using Spades and assessed with Quast against the reference genome. Left: Genome coverage. Right: Relative contig length normalized to the reference genome. K) The barcode group ratio of different species with identified plasmid reads.

To confirm that EASi‐seq generates barcoded single‐cell sequencing reads, we applied it to the synthetic ZymoBIOMICS microbial community, which consists of eight bacterial and two yeast species (**Figure** **2**A and Table S3, Supporting Information). The EASi‐seq analysis produced 205 730 606 paired‐end reads across 14 175 barcode groups after filtering for barcode read counts (Figure 2B). These barcode groups separated into two populations: 10 543 groups with low alignment rates (2.15%) and 3632 groups with high alignment rates (91.61%) (Figure 2C). We attributed the low‐alignment groups to non‐specific PCR products generated in droplets without cells, where oligonucleotides assembled on Tn5 interacted with barcode primers. In contrast, when cells were present, tagmentation products dominated amplification. Removing low‐alignment barcode groups resulted in 3632 barcode groups with an average of 38 576 reads each (range: 2000–1 931 407 reads). To assess single‐cell resolution, we mapped the reads in each barcode group to the ten reference genomes and analyzed the fraction of reads mapping to the dominant species. We found that 86.16% of barcodes had >90% purity, with most reads aligning to a single species (Figure 2D; Figure S4 and Table S4, Supporting Information). This demonstrates that EASi‐seq achieves single‐cell resolution. With shallow sequencing, the average genome coverage was 0.44% for bacteria and 0.031% for the larger yeast genomes (Figure 2E), and most barcode groups had unsaturated coverage at 10 000 reads (Figures S5 and S6, Supporting Information). Comparison with metagenomic sequencing revealed that Gram‐negative bacteria were underrepresented in EASi‐seq barcode groups (Figure 2F). Specifically, we identified 86 *S. enterica*, 46 *E. coli*, and 21 *P. aeruginosa* cells with 983 823, 788 809, and 142 191 reads, respectively. This underrepresentation aligns with prior findings^[^ ^25^ ^]^ and is attributed to the ZymoBIOMICS inactivation buffer (DNA/RNA Shield), which pre‐lyses Gram‐negative bacteria. Excluding Gram‐negative species, EASi‐seq demonstrated strong correlation with metagenomic sequencing results (Pearson correlation = 0.82, Figure 2F), indicating that EASi‐seq is robust for profiling microbial compositions.

### 2.3 Reference‐Independent Clustering of Unknown Cell Types

When applying EASi‐seq to a novel microbiome, reference genomes are usually not available for mapping and species assignment of the single‐cell datasets. Thus, to build a genome atlas that displays all cells in a sample, we require a clustering algorithm not reliant on prior knowledge of the species present. In addition, many single‐cell genomes in EASi‐seq are covered at less than 1% (Figure 2E) and lack overlapping reads across barcode groups of the same cell type. To address this, we developed the Taxonomic Discovery Algorithm (TDA), which clusters cells without requiring reference genomes by treating each barcode group as a metagenomic sample and estimating its taxonomic abundance using classifiers. In TDA, reads from each barcode group are classified based on a taxonomic database to generate genus‐level abundance vectors (Experimental Section). Each vector represents the distribution of reads mapped to various genera, proportional to the number of reads classified. Cells from related taxa generate similar vectors, even if their reads cover different genomic regions or imperfectly match the database genera. Barcode groups with nonspecific amplification products, which fail to match any taxonomy, are excluded. Using Uniform Manifold Approximation and Projection (UMAP),^[^ ^41^ ^]^ TDA clusters related cells for visualization, allowing reads within each cluster to be pooled for consensus genome assembly.

The efficacy of TDA depends on the classification method and database used. To identify the best approach, we evaluated tools such as Kraken2/Bracken,^[^ ^42^, ^43^ ^]^ MetaPhlAn3,^[^ ^44^ ^]^ and Kaiju^[^ ^45^ ^]^ using simulated microbiomes. Kraken2/Bracken with the PlusPF database (v.2021/01/27, https://benlangmead.github.io/aws‐indexes/k2)^[^ ^46^ ^]^ showed superior genus identification accuracy, barcode purity prediction, and retention rates (Discussion S1, Figure S7C–H, Supporting Information) and was chosen for subsequent analyses. Using the ZymoBIOMICS synthetic community, TDA correctly clustered all ten populations after filtering for mapped reads and genus‐level purity (Figure 2G and Table S5, Supporting Information). Furthermore, 97.34% of barcode groups were accurately annotated, reflecting good database representation of community members (Figure S8C, Supporting Information). EASi‐seq's ability to pool single‐cell data can be enhanced by integrating metagenomic sequencing, which captures nucleic acids independent of intact cells and better represents diverse taxa. Using a strategy similar to TDA, we assigned genus‐level abundance vectors to metagenomic contigs and co‐clustered them with EASi‐seq barcode groups (Figure S9 and Table S6, Supporting Information). After filtering contigs for >90% genus purity (Figure S10, Supporting Information), most clustered with single‐cell data points (Figure 2H; Figure S11, Supporting Information).

Integrating metagenomic contigs significantly increased genome coverage, achieving 94.31 ± 4.92% for bacteria and 2.74 ± 3.24% for fungi, with relative contig lengths approaching 100% of the genome (Figure 2I,J). The assembled contigs had GC content consistent with reference genomes (Figure S12A, Supporting Information) and an average N50 of 49 Kbp (Figure S12B, Supporting Information). These results demonstrate that combining metagenomic contigs with EASi‐seq enhances microbial genome capture and coverage, enabling comprehensive analysis of diverse taxa.

### 2.4 Plasmid Identification in Single‐Cell Barcode Groups

As a type of mobile genetic element, plasmids can transfer between different host bacterial cells, making it highly challenging to determine their exact host origin in a complex microbial community. Metagenomics‐based plasmid host identification typically relies on sequence alignment to known bacterial genome databases,^[^ ^47^ ^]^ GC content and other genome signature matching,^[^ ^48^ ^]^ CRISPR spacer matching,^[^ ^47^ ^]^ DNA methylation patterns,^[^ ^49^ ^]^ or other complex bioinformatic approaches, such as machine learning^[^ ^50^, ^51^, ^52^ ^]^ and hierarchical host prediction.^[^ ^53^ ^]^ These approaches are limited due to the inherent fragmentation of metagenomic data, biases in sequencing and computational methods, and the horizontal gene transfer potential of plasmids. EpicPCR allows direct linking of plasmids with host cell marker genes at the single‐cell level,^[^ ^38^, ^54^, ^55^ ^]^ but it can only be used for known, targeted plasmids. Proximity ligation techniques, such as Hi‐C^[^ ^56^, ^57^, ^58^ ^]^ or 3C,^[^ ^59^ ^]^ capture physical interactions between plasmids and host genomes, enabling direct identification of plasmid hosts. However, Hi‐C and 3C require additional experimental steps and sequencing costs and are biased toward high‐abundance genomes.

Unlike bulk metagenomics, single‐cell sequencing analyzes DNA from individual cells, enabling the direct linkage of plasmids to their host genomes. To evaluate the ability of EASi‐seq to accurately associate plasmids with their correct hosts, individual barcode groups from the ZymoBIOMICS synthetic community were aligned to five plasmid sequences from the reference genomes: one plasmid from *E. coli* (110 007 bp), one from *S. enterica* (49 572 bp), and three from *S. aureus* (6337 bp, 2216 bp, and 2993 bp, respectively). The *E. coli* plasmid and the third *S. aureus* plasmid (2993 bp) were identified exclusively within their corresponding species' barcode groups (Figure 2K). In contrast, the *S. enterica* plasmid was detected in *S. cerevisiae* and *S. aureus*, while the first two *S. aureus* plasmids (6337 bp and 2216 bp) were detected in eight species, excluding *P. aeruginosa* and *S. enterica*.

To investigate these misalignments, we hypothesized that the plasmid sequences might share similarity with other species' genomes. To test this, we calculated the average nucleotide identity (ANI) between the plasmids and the reference genome sequences. As expected, the plasmids showed 100% ANI with their corresponding species. However, each plasmid also displayed high similarity to multiple species (ANI > 73%, Figure S13, Supporting Information).

These findings suggest that sequence similarity may account for the presence of plasmid reads in other species' barcode groups. Nonetheless, their respective species exhibited significantly higher identification ratios compared to others, reinforcing a stronger association with their correct hosts despite the observed cross‐species signals (Figure 2K).

### 2.5 Strain‐Resolved Differentiation

**Figure 3:**
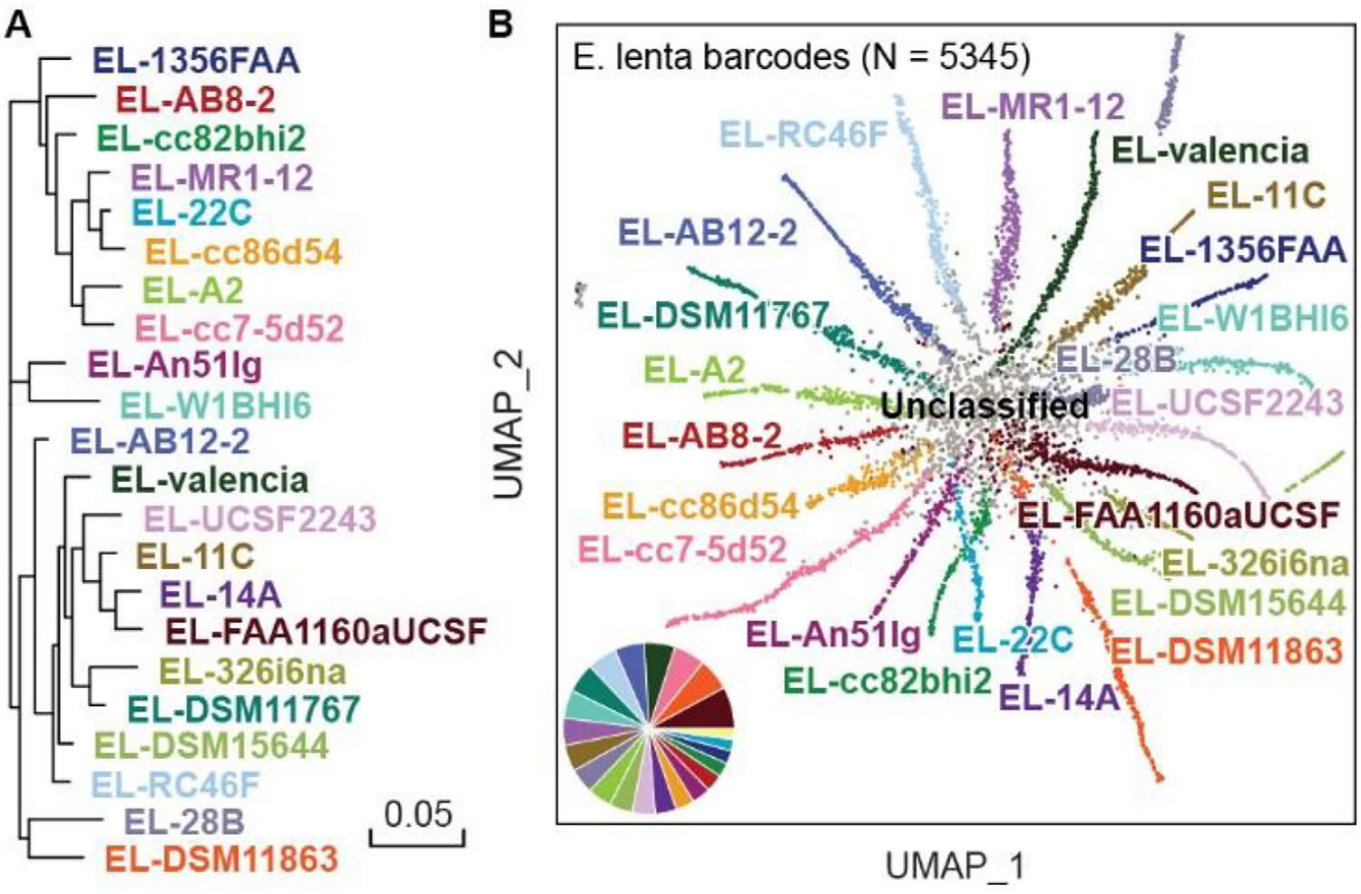
Strain resolution discrimination of microbes is achieved by EASi‐seq. A) Phylogenetic tree of the 22 *E. lenta* strains that make up the synthetic community. B) UMAP clustering based on Bayesian abundance estimation of strains in each barcode group. Colors are the same as in (A), with any mixed/unresolved barcodes colored grey. The inset pie graph quantifies the fraction of barcode counts corresponding to each strain (excluding the unclassified barcodes), showing agreement with the expected equal distribution of strains. Sample size (N) = 5345.

Differentiating between strains within a species is important for analysis of natural and engineered microbiomes.^[^ ^60^ ^]^ Because EASi‐seq can obtain thousands of reads on each cell, it affords novel opportunities for strain differentiation. To evaluate the ability of EASi‐seq to accomplish this, we used it to analyze a synthetic community consisting of twenty‐two equally mixed strains of *Eggerthella lenta* ^[^ ^61^ ^]^ (**Figure** **3**A and Table S7, Supporting Information). We sequenced the library at 105 896 184 paired‐end reads after quality filtering. We grouped the reads by barcode and aligned them against the reference genomes. To ensure read quality, we filtered barcode groups based on read counts and alignment rate (Figure S14, Supporting Information), recovering 5345 barcodes containing 101 760 151 reads. Because the strains have highly overlapping genomes, most reads align to multiple strains; thus, only reads specific to a single genome are useful for strain identification. Based on this, we developed a strain resolution approach reminiscent of transcript isoform expression estimation (BitSeq).^[^ ^62^ ^]^ We treat each genome as an isoform of one gene and estimate their “expression” level in each barcode group using BitSeq (*parseAlignment* and *estimateVBExpression* functions). All reads in a barcode group are mapped to the isoforms/strains and the probabilities of reads originating from a given isoform/strain are calculated for each alignment using a sequence‐specific bias correction method (*parseAlignment*). Alignment probabilities are then used to calculate the posterior distributions of each isoform/strain via variational Bayes inference (estimateVBExpression), which is used to determine which strain a given cell most closely resembles (Methods). We aligned the reads in each barcode group to the reference genomes and recorded the overlap, using a Log‐Normal read distribution to calculate the probability of originating from each reference genome, accounting for quality scores and mismatches. The barcode group is then assigned to a strain with more than 15% abundance and the highest abundance. (Figure S15a and Table S8, Supporting Information). To visualize the resultant annotations, we plot the data as a UMAP and pair plot of the abundance estimation (Figure 3B; Figure S16, Supporting Information). The separation between clusters on the UMAP plot confirms EASi‐seq's strain‐level resolution.

We found that 18.91% of the barcodes (1011 out of 5345, Figure 3b) were not assigned to any strain, which resulted from two factors. First, the barcodes that were not assigned to any strain typically have significantly fewer average reads (Figure S15b, Supporting Information). Second, these barcodes also exhibit fewer reads aligned to strain‐specific regions of the reference genomes. The bioinformatics pipeline used for this analysis, the parseAlignment function from BitSeq,^[^ ^62^ ^]^ relies on strain‐specific regions to estimate the probability of each strain's alignment. In barcodes with successful strain assignment, the probability score for the assigned strain is dominant, resulting in a high standard deviation across the probability scores of the 22 strains. Conversely, in barcodes without strain assignment, the standard deviation of the 22 strains’ probability scores is significantly lower (Figure S15c, Supporting Information). This observation suggests that fewer reads in these barcodes align to the strain‐specific regions, which limits the ability to confidently assign them to a particular strain.

### 2.6 Single Cell Atlas of a Human Gut Microbiome

**Figure 4:**
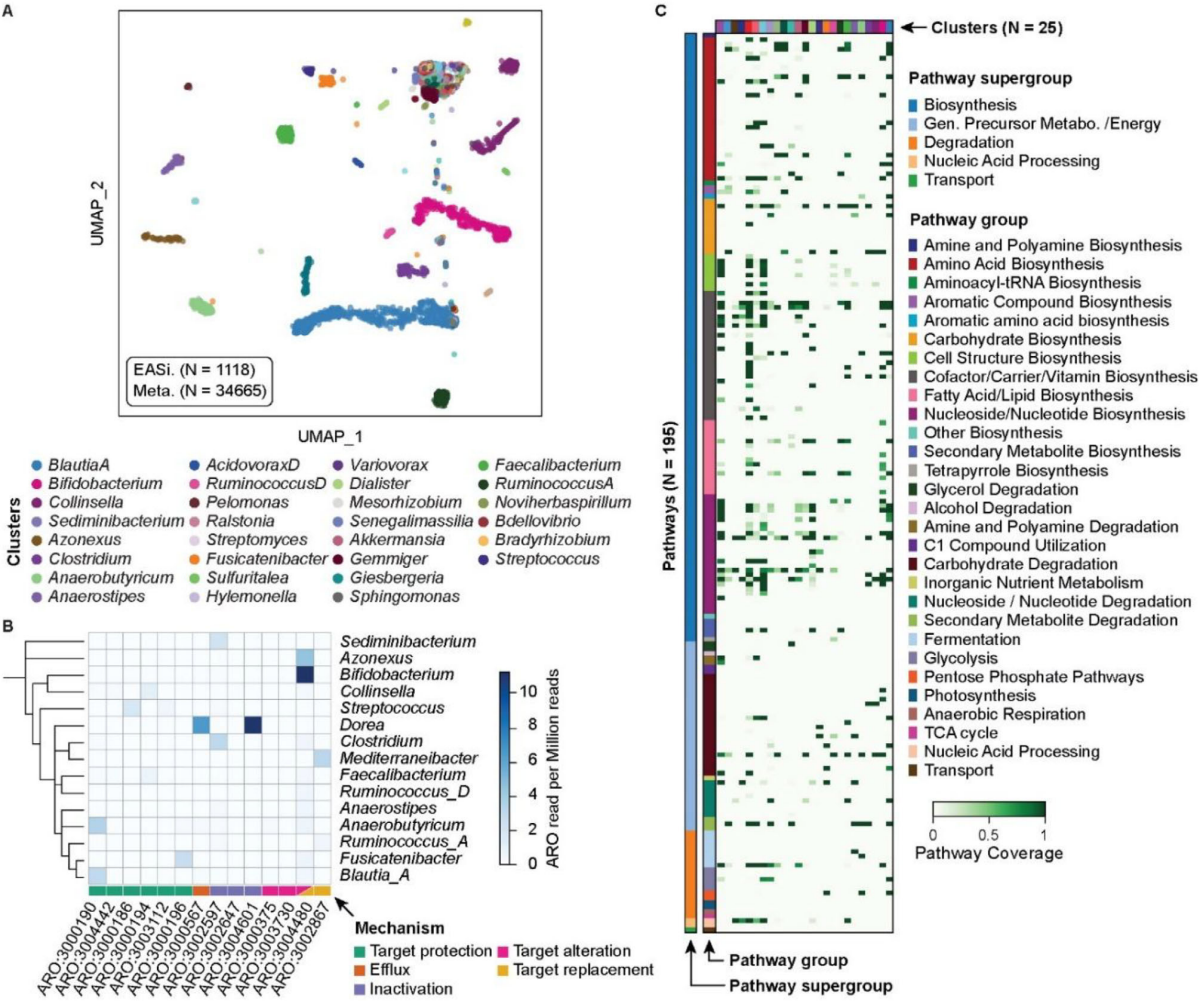
Human gut microbiome genome atlas. A) Integrated UMAP clustering of the single cell barcodes and metagenomic assembled contigs of a human microbiome sample. Each barcode/contig was annotated based on the most abundant genus. Only the top 30 clusters are labeled in the legend. Sample size for EASi‐seq (N) = 1118, Sample size for metagenomic contig cluster (N) = 34 665. B) Antibiotic resistance gene distribution in the clusters identified by TDA. Rows represented annotated genus and columns represent resistance gene access numbers from the Comprehensive Antibiotic Resistance Database. The value represents the read counts of the corresponding antibiotic resistance gene per million combined reads of each cluster. C) Relative pathway abundance in the identified clusters. All reads in EASi‐seq barcode groups associated to each cluster were combined and analyzed using MetaPhlAn and the MetaCyc database. The relative abundances of each pathway (copy per million, CPMs) were normalized to the barcode counts in each cluster. Clusters are color‐coded to the genera listed in (A).

The human gut microbiome comprises vast numbers of microbes from hundreds to thousands of species.^[^ ^63^ ^]^ Additionally, it can vary between individuals as a result of time, diet, geographical location, and health.^[^ ^64^ ^]^ Thus, characterizing microbiomes, the microbial taxa present, and their genetic functions is critical to understanding the dynamics and complexity of this ecosystem. Most approaches use amplicon sequencing of the 16S rRNA gene or bulk metagenomic sequencing.^[^ ^65^, ^66^ ^]^ EASi‐seq would provide unique information missed by these methods, including single cell‐level heterogeneity and cell‐cell interactions. To explore this possibility, cells isolated^[^ ^67^ ^]^ from the human gut microbiome of a healthy donor were profiled by EASi‐seq (Figure S17A, Supporting Information). After quality filtering, we recovered 232 705 096 paired end reads. We grouped reads by barcode and filtered by read count (>1000 reads) and genus purity estimated by Kraken2 (>80%) to remove multiplets and cell aggregates (Figure S18A,B and Discussion S2 and Table S9, Supporting Information). The recovered 1118 barcode groups contained ≈150 000 reads on average. To increase cell capture efficiency and genome coverage, we also performed metagenomic sequencing of the sample (Table S10, Supporting Information) and integrated it into the single cell data as described previously (Figures S9 and S19A–C, Supporting Information). We filtered contigs based on read percentage classified by Kraken2 and genus level purity before integration with the EASi‐seq data (Figure S19D–F, Supporting Information). We generated a cell atlas, identifying 95 clusters or microbial populations (**Figure** **4**A) with varied cell numbers and read counts (Figure S20, Supporting Information). The metagenomic data increased the number of unique reads and clustered well with the single‐cell data (Figure S22 and Table S11, Supporting Information). Nevertheless, several genera remain underrepresented in the atlas, including Bacteroides, Phocaeicola, Parabacteroides, Akkermansia, and Alistipes which may be a result of the cell isolation^[^ ^67^ ^]^ or sample storage artifacts,^[^ ^68^ ^]^ as has been described previously (Figure S21, Discussion S3, Supporting Information).

The taxonomic level of the clustering depends on the taxonomic level used for the mapping in the TDA. Since we used genus for the analysis so far, clusters in the UMAP most closely represent this level. Thus, some clusters may group cells from multiple species, which may be resolvable by isolating these groups and re‐clustering with a TDA analysis that uses species‐level Kraken2 estimation (Figure S23, Supporting Information). For example, the two clusters with the most cells (*Blautia‐A*, and *Bifidobacterium*) can be categorized into 10 and 7 sub‐clusters, respectively, corresponding to different populations of these genera coexisting in the sample.

### 2.7 Taxonomic Distribution of Antibiotic Resistance Genes

Antibiotics can profoundly impact the gut microbiome in terms of species composition, microbial metabolic activity, and antibiotic‐resistant gene (ARG) abundance.^[^ ^69^ ^]^ To evaluate ARG distribution among taxa in the fecal microbiome, we searched for ARGs in each cluster (Figure 4B and Table S12, Supporting Information) by aligning the reads against the Comprehensive Antibiotic Resistance Database (CARD).^[^ ^70^, ^71^ ^]^ We filtered the alignments by mapping score (Bowtie2 output SAM MAPQ ≥42), selected the ARGs for protein coding, and identified 14 ARGs from 15 genus clusters, with mechanisms including antibiotic target alteration, protection, replacement, inactivation, and efflux. ARGs with accession numbers ARO:3004480, ARO:3004601, and ARO:3000190 are most prevalent among the 14 ARGs and were identified in 10, 9, and 6 genus clusters, respectively. Species from *Bifidobacterium*, *Blautia_A*, *Collinsella*, and *Anearobutyricum* carry the most ARGs, at respective counts of nine, eight, six, and six. Based on those finding, we predict Bifidobacterium potentially has strong resistance (11.2 ARGs read per million reads) to rifampicin and peptide antibiotics, consistent with prior findings.^[^ ^72^ ^]^ *Dorea* also has high potential resistance to aminoglycoside antibiotic (11.1 ARGs read per million reads) and tetracycline antibiotics (6.7 ARGs read per million reads).

### 2.8 Functional Annotation of Gene Clusters Detected in Fecal Microbiome Genera

Biosynthetic pathways are often encoded as gene clusters that allow cells to acquire the ability to synthesize new molecules.^[^ ^73^, ^74^ ^]^ Many gene clusters have already been observed and characterized for function, allowing this information to be annotated to single‐cell datasets based on detection of key pathway genes, such as MetaCyc^[^ ^75^, ^76^, ^77^ ^]^ and KEGG.^[^ ^78^, ^79^, ^80^ ^]^ Using MetaCyc, in the 95 genera groupings found in our fecal microbiome, we identified 194 gene clusters belonging to 29 classes in 5 super classes, with biosynthetic functions including generation of energy precursors, degradation utilization and assimilation, transport, and macromolecule modification. Additionally, we found that different taxa possess distinct pathways, as might be expected on their unique ecological niches (Figure 4C and Table S13, Supporting Information). Even within a similar pathway type, different genera have different functions, such as amino acid metabolism. For example, *Blautia_A* possess the pathway to produce arginine, aspartate, ornithine, lysine, methionine, serine, and tryptophan; *Bifidobacterium* to synthesize the branched amino acids isoleucine, serine, and valine; *Akkermansia* to synthesize arginine, isoleucine, valine, and branched amino acid; and *Anaerobutyricum* to synthesize ornithine and methionine. Different genera also utilize distinct carbohydrate sources, with pathways for glucose, galacturonate, lactose, trehalose, sucrose, galactose, stachyose, rhamnose, and mannose all detected in the microbiome. Glucose degradation was identified in *Bifidobacterium*; sucrose degradation was seen in *Agathobacter*, *Anaerostipes*, *Coprococcus*, *Ruminococcus_D*, and *Streptococcus*; and, starchyose degradation was detected in *Blautia_A*, *Coprococcus*, *Fusicatenibacter*, *KLE1615*, and *Roseburia*. The ability to unambiguously link functional properties to community members is useful for unraveling the web of pathways that comprise all microbiomes and, ultimately, should aid in the engineering of microbiomes to improve gut health.

### 2.9 Taxonomic Distribution of Nutrient Biosynthesis Pathways

The gut microbiome is the source of vitamins and other nutrients important to health.^[^ ^81^, ^82^ ^]^ We identified 28 vitamins, cofactors, and carrier biosynthesis pathways in the fecal genome atlas, responsible for producing several vitamins and their precursors, including pantothenate (vitamin B5), adenosylcobalamin (vitamin B12), folate (vitamin B9), riboflavin (vitamin B2), thiamine (vitamin B1), biotin (vitamin B7), pyridoxal 5′‐phosphate (active form of vitamin B6), nicotinamide adenine dinucleotide (NAD) and 1,4‐dihydroxy‐6‐naphthoate (precursor of menaquinones or vitamin K2). Riboflavin is produced by 18 genera, including *Agathobaculum*, *Barnesiella*, *Parabacteroides*, and *Coprococcus*. Thiamin pathways exist in 13 genera, including *Bacteroides*, *Bifidobacterium*, *Faecalibacterium*, and *Phocaeicola*, and 19 clusters are detected for folate transformation, including, *Acetatifactor*, *Alistipes*, *Bacteroides*, *Barnesiella*, *Bifidobacterium*, *Blautia_A*, *Eubacterium_F*, *Faecalibacterium*, *Gemmiger*, *Mediterraneibacter*, *Phascolarctobacterium*, *UMGS1375*, and *Phocaeicola*. We also detected 21 clusters containing the pantothenate biosynthesis pathway, including *Acetatifactor*, *Alistipes*, *Bacteroides*, and *Gemmiger*, and that *Alistipes* also synthesizes vitamin K2. These findings show that EASi‐seq can characterize nutrient interactions between microbiome members, and between the microbiome and its host.

### 2.10 Single‐Cell Atlas of a Coastal Sea Water Microbiome

**Figure 5:**
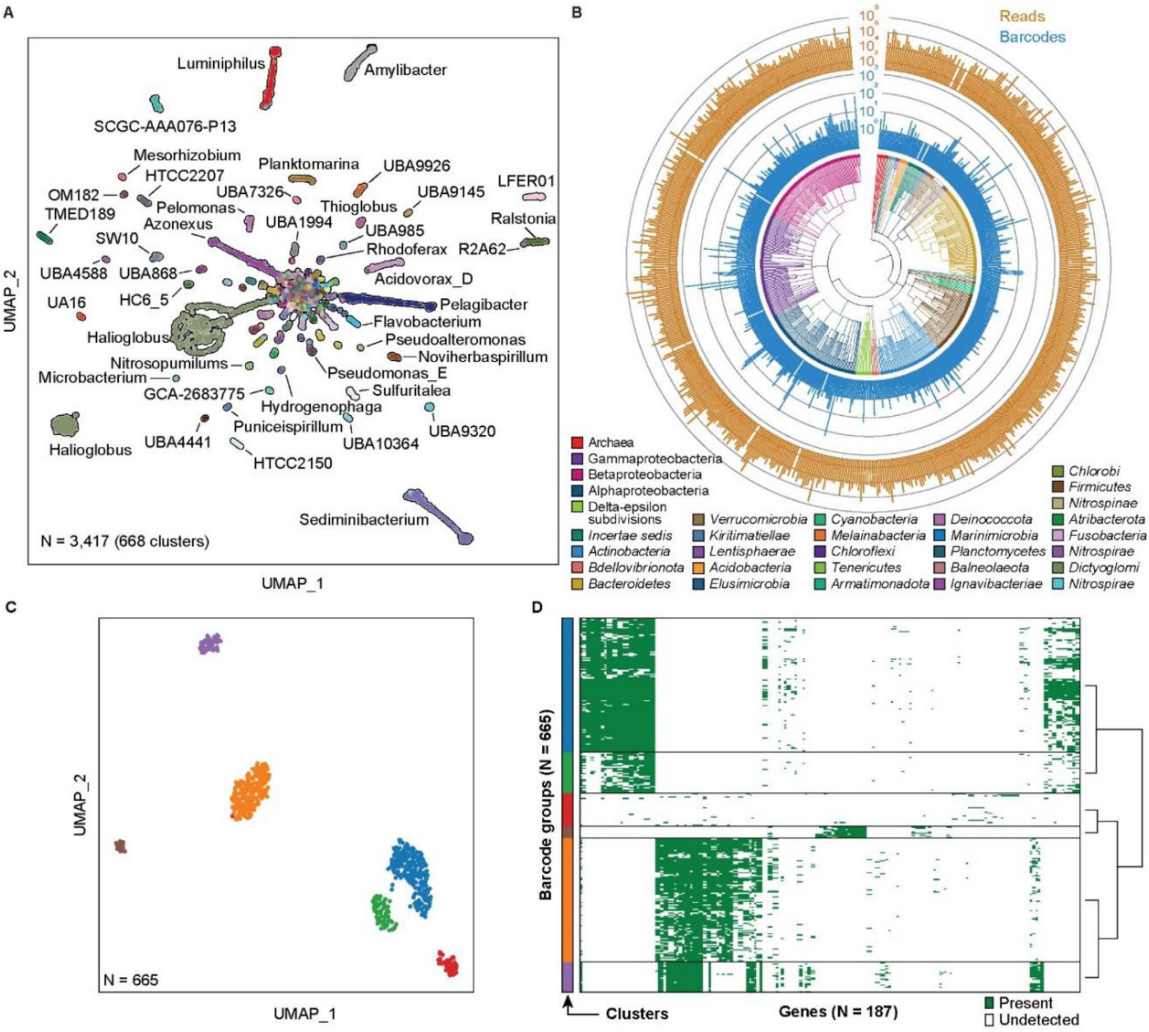
Coastal sea water microbiome genome atlas. A) UMAP based on single‐cell genera clustering, with points colored according to Kraken2 annotation. Sample size (N) = 3417. B) Phylogenetic analysis of the barcode groups. The phylogenetic tree branches are colored by phylum, except in the case of Archaea (kingdom), Gamma/ Beta/ Alphaproteobacteria (classes all part of the phylum Pseudomonadota), and incertae sedis (members with ambiguous TDA classification). The inner bar plot (blue) shows the barcode count for the corresponding genus, and the outer bar plot (gold) shows the total read counts for the corresponding genus. C) UMAP subclustering of the genus Halioglobus, from (A), which has the highest barcode count. 3873 genes are identified from the 809 barcode groups using HUMANN 3.0 with UniRef90 database. After the genes and barcode groups are filtered (minimum cell counts of a gene = 5 and minimum gene count of a cell = 5), the vector containing 665 barcode groups and 298 genes are used to generate the UMAP. D) The gene distribution in the Halioglobus barcodes. The barcodes are grouped by the cluster in (C).

Environmental microbiomes play important roles in the global ecosystem,^[^ ^83^, ^84^ ^]^ for biogeochemical cycling of elements,^[^ ^85^, ^86^, ^87^, ^88^ ^]^ metabolism of greenhouse gases,^[^ ^89^, ^90^, ^91^ ^]^ soil fertility,^[^ ^92^ ^]^ and biodegradation.^[^ ^93^ ^]^ Compared to human microbiomes, environmental microbiomes are more diverse and difficult to culture.^[^ ^94^, ^95^ ^]^ Thus, just as single‐cell atlases can reveal unique information about human microbiomes, so too can they provide insight into the microbiomes of the environment. To demonstrate the utility of EASi‐seq for analyzing environmental microbiomes, we applied it to seawater samples collected from the San Francisco coastline. We isolated the cells via filtration^[^ ^22^ ^]^ (Figure S17B, Supporting Information) and processed them with EASi‐seq to obtaining 329 470 030 paired‐end reads. Quality filtering and further filtration based on classification rate in Kraken2 (Figure S24, Supporting Information) yields 3417 cells with an average of 21 062 reads. Using the TDA, we discover 876 genus clusters (**Figure** **5**A and Tables S14 and S15, Supporting Information), of which 3395 cells are bacteria, and 22 are archaea (Figure 5B). The most abundant bacteria phyla are *Proteobacteria* (2438 cells), *Bacteroidota* (556 cells), *Actinobacteriota* (146 cells), *Verrucomicrobiota* (48 cells), *Firmicutes_A* (34 cells), and *Firmicutes* (22 cells). The archaea include *Thermoproteota* (12 cells), *Halobacteriota* (6 cells), and *Thermoplasmotota* (4 cells). To demonstrate the diversity of the captured community, we constructed a phylogenetic tree using the genus‐level identification of the cells (Figure 5B, center). Within the 668 identified genera, the top genera by abundance are *Halioglobus* (810 cells), *Sediminibacterium* (218 cells), *Pelagibacter* (190 cells), *Azonexus* (170 cells), *Luminiphilus* (154 cells), and *Amylibacter* (105 cells). This composition is consistent with previous studies of ocean microbiomes.^[^ ^22^, ^96^, ^97^ ^]^

### 2.11 Single Cell Gene Distribution in Halioglobus

To demonstrate the ability to analyze the gene distribution at single cell level, we analyzed the genus cluster with highest abundance, *Halioglobus*, which accounts for 810 barcode groups (23.7% of the 3417 total counts). The genus *Halioglobus* belongs to the class *Gammaproteobacteria* and family *Halieaceae*, which is characterized as Gram‐negative, non‐endospore‐forming, aerobic, oligotrophic, and mesophilic bacteria. The family is exclusively isolated from marine environments and is one of the major bacteria groups in coastal or open ocean environments.^[^ ^98^ ^]^ Although a few isolated *Halioglobus* species isolates have been reported,^[^ ^98^, ^99^, ^100^, ^101^, ^102^ ^]^ the heterogeneity within a *Halioglobus* population has never been studied. Within the single‐cell cluster, we first annotate genes using HUMAnN3.^[^ ^44^ ^]^ After filtering based on the gene count and cell counts (minimum cell counts per gene = 10 and minimum gene counts per cell = 10), the gene presence/absence matrix of the *Halioglobus* barcode groups are grouped into 6 clusters by Leiden algorithms^[^ ^103^ ^]^ (Figure 5C). The gene presence/absence matrix of each cluster is shown in Figure 5D. This result suggests the *Halioglobus* genus in our sample is a heterogeneous population and indicates that EASi‐seq is suitable for the analysis of a heterogeneous population, which could potentially be used for more detailed single‐cell‐resolution pan‐genome analysis.

## 3 Discussion

Microbes play key roles in all ecosystems and are important to human health. While they comprise the most diverse forms of life on the planet^[^ ^104^ ^]^ there are few tools available for sequencing them at single‐cell resolution. Additionally, while tools for single mammalian cell genomics have become widespread,^[^ ^105^ ^]^ analogous tools for microbes have lagged, due to the technical challenges of isolating and sequencing them in the numbers required to characterize diverse microbiomes. With these realizations in mind, we developed a workflow for efficient microbe sequencing using Mission Bio's commercial single‐cell platform. This instrument is broadly distributed and accessible to non‐experts, and therefore constitutes an opportunistic foundation on which to build a single microbe sequencing technology. A major element enabling this was our development of a simple and general bulk technique to purify single‐cell genomes in hydrogels that are compatible with the instrument. Our lysis procedure is applicable to all microbe types, including archaea, bacteria, and fungi, and the commercial microfluidics allow high throughput and efficient single‐cell barcoding, to obtain unbiased sequencing for tens of thousands of cells in a sample per run.

The data generated by EASi‐seq is unique in that reads are grouped at the level of single cells. By contrast, the dominant method of metagenomic sequencing discards single‐cell information and captures the sequence data as a mixed pool of short reads. This mixed pool output necessitates complex bioinformatic approaches for strain variation analysis or contig reconstruction that cannot exploit single‐cell information. EASi‐seq not only overcomes these limitations, but also provides unique methods validating bioinformatic algorithms by comparing results with EASi‐seq datasets.^[^ ^106^ ^]^ In addition to developing a novel approach for obtaining single‐cell data, we also develop novel bioinformatic approaches that exploit the data's single‐cell structure. These include ways to allow cells to be clustered by similarity, aggregation of the reads within a cluster to increase genome coverage, annotate phylogeny and genes, and to scan genomes for genetic elements of interest. By enabling the construction of detailed cell atlases that capture the overall species demographics of a microbiome, EASi‐seq affords new opportunities for characterizing the interaction webs inherent to these systems that are near impossible to obtain with metagenomic techniques. With the ability to integrate EASi‐seq and metagenomic reads, EASi‐seq can provide a complementary viewpoint to metagenomics.

There still remain aspects of the EASi‐seq method that can be improved. Importantly, the coverage per barcode is low, which is caused by three reasons. First, before droplet barcoding, the genomic DNA is fragmented by Nextera‐like tagmentation, which leads to only 50% of the genomic fragments being viable for barcoding PCR.^[^ ^40^ ^]^ To overcome this inefficiency, we anticipate that future implementations of EASi‐seq can increase the complements of adaptors^[^ ^107^ ^]^ or use single‐adaptor transposition and uracil‐based adapter switching within the barcoding PCR step.^[^ ^108^ ^]^ Second, the heterogeneous genome sizes of microbes require different amounts of transposase to achieve the appropriate fragment size.^[^ ^109^ ^]^ Although we did extensive optimization, one concentration does not fit all needs. For certain genomes, the transposase concentration could either be too high (for smaller genomes) and generate fragments that are below the size‐selection threshold or too low (for larger genomes) and produce long fragments incompatible with downstream processing. Third, in adapting the protocol to directly integrate into a commercial device, it was necessary to utilize the barcoding beads from the Tapestri V2 reagent kit. The beads’ barcoding primer has a 15 bp constant region with a melting temperature of 48 °C. While we used this sequence as the forward priming site in the barcoding PCR, a higher temperature of 55 °C was used as the anneal temperature to avoid random priming. This may lower efficiency in the PCR step. Future optimization can involve development of barcoding beads with improved primers having an elevated melting temperature. Finally, we suspect that coverage can be also improved with an additional single genome amplification step prior to the tagmentation, which can be achieved either in droplet^[^ ^23^ ^]^ or in hydrogel beads.^[^ ^15^ ^]^ Such improved coverage will greatly advance the application of EASi‐seq.

Even in its current form, EASi‐seq represents a highly accessible platform technology for generating detailed and comprehensive single‐cell genome atlases independent of isolation and culturing. Such atlases will have a broad and sustained impact on microbiology, similar to what has been accomplished for mammalian cells. Because we build our workflow on a commercial architecture that is constantly adding features, many of the same improvements and innovations may carry over to microbiomes. For example, after the first demonstrations of mammalian cell DNA and RNA sequencing, multiomic approaches were built on top of the original technologies. These include the ability to measure surface and internal proteins, characterize epigenetic signatures and genome structure, and integrate spatial data.^[^ ^110^ ^]^ For example, microbial RNA‐seq is possible using universal cDNA methods amenable to single‐cell barcoding and would thus allow the addition of transcriptional state measurements with EASi‐seq. Using oligonucleotide‐labeled binders like including antibodies, lectins, and aptamers, microbes can be stained prior to EASi‐seq, allowing for recording proteomic and serotype signatures in a manner similar to Ab‐seq,^[^ ^111^ ^]^ DAb‐seq,^[^ ^112^ ^]^ CITE‐seq,^[^ ^113^ ^]^ inCITE‐seq,^[^ ^114^ ^]^ and INS‐seq.^[^ ^115^ ^]^ Similarly, the lysis and molecular biology processes of EASi‐seq should carry over to DNA viruses and, with the implementation of reverse transcription, RNA viruses, holding potential for single virus genome atlas.

## 4 Experimental Section

### Microbiome Samples Processing—Synthetic community

ZymoBIOMICS standard (Zymo, D6300) was stored at −80 °C until use. 100 µL of ZymoBIOMICS was washed with 4 mL of PBS for three times to remove the storage buffer. The cell density was measured with Countess cell counting slides (Thermo Fisher, C10228) using an EVOS microscope. After counting, cells were resuspended to a final concentration of 100 million per mL in PBS.

All 22 E. Lenta strains (Table S3, Supporting Information list of E. lenta strains) were cultured in appropriate media^[^ ^61^ ^]^ and equally mixed based on CFU counting in culture media. The cell mixture was stored at −80 °C until use. Before processing, thawed cells were washed three times to remove the storage media and filtered with 5 µm syringe filter to remove cell aggregates. After cell counting, the cells were resuspended to 100 million per mL in PBS.

### Microbiome Samples Processing—Human Microbiome and Cell Isolation

Fecal sample from health donor was stored at −80 °C until use. Cell isolation was performed according to previously reported protocol.^[^ ^67^ ^]^

### Microbiome Samples Processing—Ocean Water Microbiome and Cell Isolation

Sea water was collected at the Pacific coastline near San Francisco (GPS coordinate: 37.7354373 N, 122.5081862 W) by submerging a 1000 mL sterile bottle into the ocean. The sea water was transferred to the lab on ice. The cells were enriched according to the published protocol.^[^ ^22^ ^]^ Briefly, the sea water was first filtered through a 50 µm cell strainer (Corning, 431752) to remove sands or other large particles. The suspension was then filtered by a 0.45 µm vacuum filter (Millipore, SCHVU01RE) to capture the cells on the membrane. The membrane was cut off from the filter with a sterile razor blade and transferred a 15 mL centrifuge tube with 5 mL PBS. The cells were released from the membrane by vortexing the tube at maximum speed for 2 min. The cells were washed with 10 mL PBS for three times and passed through a 5 µm syringe filter to remove remaining virus or large particles. The cells were resuspended to 100 million per mL in PBS.

### Microfluidics Device Fabrication

Microfluidics devices were fabricated with standard photolithography and soft lithography method. Custom device fabrication was not necessary for the single‐cell sequencing using Mission Bio Tapestri but used for workflow optimization. Master photomask was designed using AutoCAD and printed at 12 000 DPI (CAD/Art Services, Bandon, OR). To make the master structure, SU8 Photoresist (MicroChem, SU8 3025 and SU8 3050) were spin‐coated on three‐inch silicon wafers (University Wafer), soft baking at 95 °C for 10–20 min, UV‐treated through the photomasks for 3 min, hard baked at 95 °C for 5 to 10 min and developed in propylene glycol monomethyl ether acetate (Sigma Aldrich). For the microfluidic devices, poly(dimethylsiloxane) (PDMS) (Dow Corning, Sylgard 184) and curing agent were mixed in 10:1 ratio, degassed and poured over the master structure, baked at 65 °C for 4 h to cure, and peeled off from the wafer. After hole punched with a 0.75 mm biopsy puncher, the devices were plasma treated and bonded to glass slides. The channels were treated with Aquapel (PPG industry) to for hydrophobic surface and dried by baking at 65 °C for 10 min.

### Single Cell Genomic DNA Isolation in Hydrogel Beads—Cell Encapsulation in Hydrogel Beads

500 µL cell suspension (100 million per mL in PBS) was mixed with 500 µL hydrogel precursor solution (12% acrylamide, 1% BAC, 20 mm Tris, 0.6% sodium persulfate, and 20 mm NaCl in H2O) in a 15 mL centrifuge tube. 1 mL HFE 7500 with 2% surfactant (008‐FluoroSurfactant, RanBiotechnologies) was added to the cell/hydrogel precursor mixture. Emulsion was formed by passing the oil/aqueous mixture five times through a 20‐gauge syringe needle. 20 µL of TMEDA (tetramethylethylenediamine, Sigma) was added into the emulsion and the emulsion was incubated at 70 °C for 30 min and at room temperature for overnight for gelation. The emulsion can be stored at 4 °C for up to 1 week.

The emulsion was centrifuged at 1000 RCF for 1 min and the bottom oil layer was removed by using a gel loading tip. 1 mL of 20% PFO (1H,1H,2H,2H‐perfluoro‐1‐octanol, Sigma, 370533) and 5 mL of PBST buffer (0.4% tween 20 in PBS) were added into the emulsion. The mixture was vortexed at maximum speed for 1 min to break the emulsion and centrifuged at 1000 RCF for 5 min. Any remaining oil was removed by pipetting through a gel‐loading tip.

### Microbiome Samples Processing—Hydrogel Size Selection

Differential velocity centrifugation was performed to select the hydrogel beads from previous step within the diameter between 5 and 15 µm. 500 µL hydrogel beads were resuspended in 14 mL high‐density buffer (40% sucrose in PBS with 0.4% tween 20). First, the beads were centrifuged at 1000 RCF for 5 min to pellet large gels. The supernatant was transferred to a new 15 mL tube and centrifuged at 3000 RCF for 10 min to pellet the right‐sized beads. The supernatant (still containing beads smaller than 5 µm) was discarded and the pelleted beads were washed 3 times with PBST to remove the high‐density buffer. Typically, ≈300 µL of size‐selected beads can be recovered.

### Microbiome Samples Processing—Cell Lysis in Hydrogel Beads

100 µL of size selected beads were treated in 1 mL cell wall digestion buffer (TE buffer solution containing 2.5 mm EDTA, 10 mm NaCl, 2U zymolyase, 5 U Lysostaphin, 50 U mutanolysin, and 20 mg Lysozyme) at 37 °C overnight. The beads were then pelleted by centrifugated at 3000 RCF for 10 min and washed with PBST for three times. The beads were then treated in 1 mL protein digestion solution (TE buffer with 4U of Proteinase K, 1% triton X100 and 100 mm of NaCl) at 55 °C for 30 min. Following lysis, the beads were thoroughly washed with PBST, 100% EtOH, and PBST three times to ensure complete removal of proteinase K and other chemicals that may inhibit the downstream reactions. The beads were then filtered with 10 µm cell strainer and ready for droplet tagmentation.

### Single Cell Tagmentation and Barcoding in Droplet Microfluidics

Microfluidic droplet encapsulation, tagmentation, and barcoding PCR were performed on commercial single‐cell DNA genotyping platform (Mission Bio, Tapestri) or custom build microfluidic devices with the same functions.

### Single Cell Tagmentation and Barcoding in Droplet Microfluidics—Tagmentation Reagents

25 µL Tn5‐Fwd‐oligo GTA CTC GCA GTA GTC AGA TGT GTA TAA GAG ACA G (100 nm, IDT), 25 µL, Tn5‐Rev‐oligo TAC CCT TCC AAT TTA ACC CTC CAA GAT GTG TAT AAG AGA CAG (100 nm, IDT), and 25 µL Blocked ME Complement /5Phos/C*T* G*T*C* T*C*T* T*A*T* A*C*A*/3ddC/ (200 nm, IDT) and 25 µLTris buffer were mixed well in a PCR tube by pipetting. The mixture was incubated on a PCR thermal cycler with the following program: 85 °C for 2 min, cools to 20 °C with a ramping rate at 0.1 °C s^−1^, 20 °C for 1 min, then held at 4 °C with lid at 105 °C. 100 uL of glycerol was added into the annealed oligo. Unloaded Tn5 protein (1 mg mL^−1^, expressed by QB3 MacroLab, Berkeley, CA), dilution buffer (50% Glycerol, 100 mm NaCl, 0.1 mm EDTA, 1 mm DTT, and 0.1% NP40 in 50 mm Tris‐HCl pH 7.5 buffer), and the pre‐annealed adapter/glycerol mix were mixed at 1:1:2 ratio by pipetting. The mixture was incubated at room temperature for 30 min then stored at −20 °C until use. For droplet tagmentation, equal amount of assembled Tn5 and tagmention buffer (10 mm MgCl2, 10 mm DTT in 20 mm TAPS pH 7.0 buffer) were mixed.

### Single Cell Tagmentation and Barcoding in Droplet Microfluidics—Droplet Tagmentation

In the first droplet step, the tagmentation reagents (0.125 mg mL^−1^ assembled Tn5, 10 mm MgCl2, and 10 mm DTT in 20 mm TAPS pH 7.0 buffer) and the genomic DNA in hydrogel beads (equivalent to three million cells per mL) in 10 mm MgCl2, 1% NP40, 17% Optiprep, and 20 mm TAPS pH 7.0 buffer were co‐flowed in the microfluidic devices to form droplets.

In case of using Tapestri, the MissionBio Tapestri DNA cartridge and a 0.2 mL PCR tube were mounted onto the Tapestri instrument. 50 µL beads solution, 50 µL tagmentation reagents, and 200 µL encapsulation oil were load in the cell well (reservoir 1), lysis buffer well (reservoir 2), and encapsulation well (reservoir 3) on the Tapestri DNA cartridge, respectively. The Encapsulation program was used for droplet generation. The droplets were collected into a PCR tube.

For custom build microfluidic device, the beads solution, the tagmentation reagents, and 5% (w/w) PEG‐PFPE surfactant (Ran Biotechnologies) in HFE 7500(3 m) were loaded into three syringes and placed on syringe pumps. The syringes were connected to the co‐flow droplet generator device via PTFE tubing. The pumps were controlled by a Python script (https://github.com/AbateLab/Pump‐Control‐Program) to pump bead solution at 200 µL h^−1^, tagmentation reagents at 200 µL h^−1^ and oil at 600 µL h^−1^ to generate droplets. The droplets were collected into PCR tubes.

The droplets generated by either method were incubated at 37 °C for 1 h, 50 °C for 1 h, and held at 4 °C to ensure hydrogel melting and Tn5 complete reacting.

### Single Cell Tagmentation and Barcoding in Droplet Microfluidics—Droplet Barcoding PCR

The tagmentation droplets from the previous were merged with PCR reagents and barcode beads for barcoding with either Tapestri or custom build microfluidic devices.

In case of using Tapestri, 8 PCR tubes and DNA cartridge were mounted onto the Tapestri instrument. Electrode solutions were loaded into electrode wells (200 µL and 500 µL in reservoirs 4 and 5, respectively). After running the Priming program, 5 µL of reverse primer (GTC TCG TGG GCT CGG AGA TGT GTA TAA GAG ACA GTA CCC TTC CAA TTT AAC CCT CCA, 100 µm, IDT) was mixed with 295 µL Mission Bio Barcoding Mix and loaded into PCR reagent well (reservoir 8) of the DNA cartridge. The droplets from previous step (≈80 µL), 200 µL of V2 barcoding beads, and 1.25 mL of Barcoding oil were loaded into cell lysate well (reservoir 6), barcode bead well (reservoir 7) and barcode oil well (reservoir 9), respectively. The droplets were merged with barcoding beads and PCR reagents by the Cell Barcoding program. The resulting droplets were collected into the 8 PCR tubes.

In case of using custom build microfluidics, the device was first primed by filling electrode solution (2 m NaCl solution) into the electrode and the moat channels. 500 µL PCR reagents containing 1.67X Q5 High‐Fidelity Master Mix (NEB, M0515), 0.625 mg mL^−1^ BSA, 1.2 µm reverse primer (GTC TCG TGG GCT CGG AGA TGT GTA TAA GAG ACA GTA CCC TTC CAA TTT AAC CCT CCA) were loaded into a 1 mL syringe. 200 µL Mission Bio V2 barcoding beads washed with Tris buffer (pH 8.0) and resuspended in 10 mm Tris buffer containing 3.75% tween 20, 2.5 mm MgCl_2_, 0.625 mg mL^−1^ BSA. The beads were centrifuged at 1000 RCF for 1 min and the supernatant was removed. The remaining bead slurry (∼110 uL) was loaded into PTFE tubing connected to a 1 mL syringe filled with HFE 7500 oil. The droplets after tagmentation were loaded into a 1 mL syringe. The three syringes and two syringes filled with 10 mL of 5% (w/w) PEG‐PFPE surfactant (Ran Biotechnologies) in HFE 7500(3 m) HFE 7500 were connected to the microfluidic devices. The following rates are as follows: tagmenation droplets 55 µL h^−1^, spacer oil 200 µL h^−1^, PCR reagent 280 µL h^−1^, barcode beads 148 µL h, and droplet generation oil 2000 µL h^−1^. To merge the tagmentation droplet, the electrode near the droplet generation zone was charged with an alternating current (AC) voltage (3 V, 58 kHz). And the moat channel was grounded to prevent unintended droplet coalescence at other locations on the device. The merged droplets were collected into PCR tubes.

The droplets collected in the merging step were treated with UV for 8 min (Analytik Jena Blak‐Ray XX‐15L UV light source) and the bottom layer of oil in each tube were removed using a gel loading tip to leave up to 100 µL of droplets. The tubes were placed on PCR instrument and thermo‐cycled with the following program: 10 min at 72 °C for 1 cycle, 3 min at 95 °C for 1 cycle, (15 s at 95 °C, 15 s for 55 °C, and 2 min at 72 °C) for 20 cycles, and 5 min at 72 °C for 1 cycle with the lid set at 105 °C.

### Single Cell Tagmentation and Barcoding in Droplet Microfluidics—Barcoded Amplicon Purification

The thermal cycled droplets in the PCR tubes were carefully transferred into two 1.5 mL centrifuge tubes (equal amount in each). If there were visible merged large droplets present, they were carefully removed using a 2 µL pipette. 20 µL PFO were added into each tube and mixed well by vortex. After centrifuging at 1000 RCF for 1 min, the top aqueous layers in each tube were transferred into new 1.5 mL tubes without disturbing the bead pellets and water was added to bring the total volume to 400 µL. The barcoding product was purified using 0.7X Ampure XP beads (Beckman Coulter, A63882) and eluted into 50 µL H2O and stored at −20 °C until next step. The concentrations of the barcoding product were measured with Qubit 1X dsDNA Assay Kits (ThermoFisher, Q33230).

### Barcoding Sequencing Library Preparation and Sequencing—Library Prep and QC

The sequencing library were then prepared by attaching P5 and P7 sequences to the barcoding products using Nextera primers (Table S2, Supporting Information). The library PCR reagents containing 25 uL Kapa HiFi Master mix 2X, 5 uL Library P5 index primer (4 um), 5 uL Library P7 index primer (4 um), 10 uL purified barcoding products (normalized to 0.2 ng uL^−1^), and 5 uL of nuclease‐free water were thermal cycled with the following program: 3 min at 95 °C for 1 cycle, (20 s at 98 °C, 20 s for 62 °C, and 45 s at 72 °C) for 12 cycles, and 2 min at 72 °C for 1 cycle. The sequencing library was purified with 0.69X Ampure XP beads and eluted into 12 uL nuclease‐free water. The library was quantified with Qubit 1X dsDNA Assay Kits and DNA HS chips on bioanalyzer or D5000 ScreenTape (Agilent, 5067‐5588) on Tapestation (Agilent, G2964AA). The libraries were pooled and 300 cycle pair‐end sequenced by Illumina MiSeq, NextSeq, or NovaSeq platform.

### Sequencing File Barcode Extraction and Single Cell Read File Preparation

Raw sequencing FASTQ files were processed using a custom python script (mb_barcode_and_trim.py) available on GitHub (https://github.com/AbateLab/MissonBioTools) for barcode correction and extraction, adaptor trimming, and grouping by barcodes. For all reads, combinatorial cell barcodes were parsed from Read 1, using Cutadapt (v2.4),^[^ ^116^ ^]^ and matched to a barcode whitelist. Barcode sequences within a Hamming distance of 1 from a whitelist barcode were corrected. Reads with valid barcodes were trimmed with Cutadapt to remove 5′ and 3′ adapter sequences and demultiplexed into individual single‐cell FASTQ files by barcode sequences using the script demuxbyname.sh from the BBMap package (v.38.57).^[^ ^117^ ^]^

### Reference Based Single Cell Data Analysis—ZymoBIOMICS Microbial Community Standards

The reference genome FASTA files of the ten species of Zymo BIOMICS Microbial Community Standards provided by Zymo Research Corporation (https://s3.amazonaws.com/zymo‐files/BioPool/ZymoBIOMICS.STD.refseq.v2.zip). The FASTA files were combined and Bowte2 index were built using Bowtie2‐build command. The reads in single‐cell FASTQ files were aligned to reference genomes using Bowtie2 (v 2.3.5.1) with default setting.^[^ ^118^ ^]^ The overall alignment rates for each barcode were collected from the log files. The barcode groups less than 50% overall coverage rate were removed. Each barcode group's coverages, numbers of mapped reads, covered bases, and mean depths of 10 corresponding species were calculated using Samtools v1.12 (*samtools coverage*) with default setting.^[^ ^119^, ^120^ ^]^ The purity of each barcode group was calculated as the percentage of reads that aligned to a dominant species. For the rarefaction analysis, 10 000 reads were randomly sampled from the SAM file of each barcode group. The coverage was calculated after each read sampling using Samtools.

### Reference‐Based Single Cell Data Analysis—Strain Abundance Estimation for Synthetic Community with 22 E. Lenta Strains

The reference genomes of the 22 E. lenta strains were downloaded from NCBI (Table S3, Supporting Information). The reads in single‐cell FASTQ files were aligned to reference genomes using Bowtie2 (v 2.3.5.1)^[^ ^118^ ^]^ with ‐a setting to report all matches. The overall alignment rates for each barcode were collected from the log files. The barcode groups with less than 50% overall coverage rate were removed. The probabilities of each alignment were calculated with *parseAlignment* command from BitSeq (v 1.16.0).^[^ ^62^ ^]^

### Taxonomic Discovery Algorithm—TDA Validation Using Simulation Data

100 species were randomly selected from the NCBI assembly metadata file (https://ftp//ftp.ncbi.nlm.nih.gov/genomes/genbank/bacteria/assembly_summary.txt). The reference genome FASTA files were downloaded using the corresponding link in the metadata file (Table S4, Supporting Information). Simulated pair‐end read files were generated using a Python script according to the following rules. 1) 100 barcode groups were generated for each species. 2) The reads were 150 bp paired end. 3) The amplicon length was in the range of 400–1000 bp. 4) Each barcode group had 0–49% percent of contamination reads. 5) The contamination reads were generated from the other 99 species. 6) Each barcode had 1000–10 000 pair‐end reads.

3 taxonomic classifiers were chosen for evaluation: Kraken2/Bracken^[^ ^42^, ^43^ ^]^ with PlusPF database (https://benlangmead.github.io/aws‐indexes/k2, Version: 1/27/2021), Kaiju^[^ ^45^ ^]^ with its standard database and MetaPhlAn 3^[^ ^44^ ^]^ with its standard database. All the pair‐ended barcode group FASTA files were profiled using the three classifiers. The results were grouped and analyzed in Python. The predicted taxa purity was the abundance of the dominant taxa in each barcode group. The barcode filtering based on purity was performed using thresholds ranging from 50% to 99% purities.

The average after‐filtering purity was the mean purity of all the barcodes that passed a certain threshold and after‐filtering barcode counts was the barcode count of that passed a certain threshold. The UMAP clustering was performed with the genus abundances of all the barcode groups. The identity of each cluster was assigned with the most abundant taxa. The identification accuracy was calculated as the percentage of barcodes with the correct genus identification.

### Taxonomic Discovery Algorithm—TDA Analysis of Single Cell Sequence Data

The single‐cell sequencing barcode group FASTQ files of ZymoBIOMICS, Human microbiome, and the sea water microbiome samples were analyzed using TDA with Kraken2/Bracken as the taxonomic identifier. For the Zymo BIOMICS sample, Kraken2 PlusPF database (https://benlangmead.github.io/aws‐indexes/k2, Version: 1/27/2021) was used, while for human microbiome and sea water microbiome, Kraken2 GTDB database (https://gtdb.ecogenomic.org/tools, Release 95) was used. The reads in each barcode group were first classified by Kraken2, and the abundances at genus and species level were re‐estimated with Bracken using default threshold setting. The percentages of the mapped reads were extracted from the Kraken2 output files of barcode groups. The purities were calculated as the abundance of the dominant genus in the barcode groups. The data was filtered according to percentage of mapped reads and genus‐level purity. The taxa abundance profiles of the remaining barcodes were combined and UMAP clustering was performed using The Scanpy toolkits^[^ ^121^ ^]^ in Python script. The taxa of each barcode group were assigned to the most abundant one.

### Metagenomic Sequencing and Assembly—ZymoBIOMICS Community

The metagenomic sequencing data of ZymoBIOMICS Microbial Community Standards D6300 (batch ZRC195925) was provided by Zymo Research Corporation. The reads were assembled using SPAdes‐3.15.3 with “–meta” setting.^[^ ^122^ ^]^

### Metagenomic Sequencing and Assembly— Human Microbiome

The human fecal sample was collected from a healthy adult donor under a UCSF IRB‐approved protocol (#14‐13821). The sample was deposited into a commode specimen collection system and aliquoted into 2 mL cryovials with DNA/RNA shield (Zymo). For bulk metagenomic sequencing, the sample was extracted using the ZymoBiomics 96 MagBead DNA kit. The sequencing library was prepared using the Nextera XT protocol and sequenced using an Illumina Nova‐Seq with 2×140 chemistry at the Chan Zuckerberg Biohub (San Francisco, CA). Metagenomic reads were quality‐filtered using FastP (v. 0.20.0).^[^ ^123^ ^]^

### Metagenomic Sequencing and Assembly—Comparison Between Metagenomic and Single Cell Sequencing

The genus abundances of the human microbiome metagenomic data and the pooled single‐cell sequence file were analyzed using Kraken2 and Bracken. The results were plotted as a scatter plot with triangle markers. For any genus with a barcode group associated, a round marker of the genus was added and its size was proportional to the barcode counts.

### Single Cell Sequencing Data Integration with Metagenomics

To integrate the metagenomic dataset, the contigs assembled from metagenomic sequencing (ZymoBIOMICS and human microbiome sample) were treated as individual barcodes and processed with TAD. The metagenomic reads were first aligned to the assembled contigs using Bowtie2 v2.3.5.1.^[^ ^118^ ^]^ The metagenomic barcode data table and the single‐cell barcode data table, containing relative abundances at the genus level, were then merged using the *ingest* function in the Scanpy toolkit (*scanpy.tl.ingest*)^[^ ^121^ ^]^ in Python. To enable merging with the ingest function, both datasets needed to have the same variables (genera). Therefore, the union of genera from both datasets was included, and zero values were assigned to empty entries prior to data integration.

### Clustered Barcode Groups Analysis—Cluster Assembly and Evaluation

Single‐cell barcodes of UMAP clusters were combined using concatenate command (cat) in the Linux system into single FASTQ files. The pair‐end reads associated with barcodes that belong to the same UMAP clusters were grouped by Seqtk toolkit (https://github.com/lh3/seqtk) (*seqtk subseq*) into single FASTQ files. The assemblies were conducted with all reads associated to both single‐cell sequencing and metagenomic contigs of each UMAP cluster using Spades v 3.15.3^[^ ^122^ ^]^ with “–careful” setting. The assembled contigs were evaluated using Quast v 5.0.2^[^ ^124^ ^]^with or without reference genome input. To calculate the clustering error rate, all the reads associated to a cluster were mapped to the corresponding reference genome, the percentage of the reads that were not aligned was considered as the error rate.

Pathway analyses of each cluster was conducted using HUMAnN v 3.0^[^ ^44^ ^]^ with the default MetaCyc^[^ ^75^, ^77^, ^125^ ^]^ database. The pathway abundance files of each cluster were combined and plotted as a heatmap using the Seaborn module in Python.

The sub‐categorizing of barcode groups in a UMAP cluster was using species abundance estimation. The two clusters with the most barcode groups in the human microbiome samples (*Blautia_A*, and *Bifidobacterium*) were further divided into subclusters by UMAP aggregation with the Kraken2 species abundance estimation.

### Clustered Barcode Groups Analysis—Gene Association Analysis

Comprehensive Antibiotic Resistance Database (CARD) (v 3.1.4)^[^ ^70^ ^]^ (https://card.mcmaster.ca/download) was downloaded and bowtie2 references were built with botie2‐build command.^[^ ^118^ ^]^ The combined reads associated with each UMAP cluster identified in the human gut microbiome were mapped to the CARD databases using Bowtie2 (v2.3.5.1).^[^ ^118^ ^]^ The mapping reads were filtered for MAPQ ≥ 42 to select the reads without mismatches using SAMTools (samtools view ‐bS ‐q 42).^[^ ^119^, ^120^ ^]^ After duplicate reads were removed using SAMTools (samtools rmdup ‐S),^[^ ^119^, ^120^ ^]^ the references sequence name (RNAME) of each alignment were extracted from the bam files. The unique genes associated with each UMAP cluster, and their frequencies were generated from the RNAMEs. The relative abundance antibiotic resistance gene was calculated as the unique ARO read count per million total read count. The resistance mechanism associated with antibiotic resistance ontology (AROs) were downloaded from the Comprehensive Antibiotic Resistance Database.

### Clustered Barcode Groups Analysis—Plasmids Search in Barcode Groups

The reference genome FASTA files for the ten species included in the ZymoBIOMICS Microbial Community Standards were provided by Zymo Research Corporation (https://s3.amazonaws.com/zymo‐files/BioPool/ZymoBIOMICS.STD.refseq.v2.zip). Plasmid reads were saved as a separate FASTA file, and Bowtie2 indices (*bowtie2‐build*) for the plasmids were generated using the bowtie2‐build command. Reads from the single‐cell FASTQ files were aligned to the reference genomes using Bowtie2 (v2.3.5.1) with default settings.^[^ ^118^ ^]^ The aligned reads were filtered for a mapping quality score (MAPQ) of ≥42 to select reads without mismatches, using SAMtools (*samtools view ‐bS ‐q 42*).^[^ ^119^, ^120^ ^]^ The binary results indicating plasmid presence in each barcode group were then merged with the species assignments from “ZymoBIOMICS Microbial Community Standards.” The ratios of barcodes containing plasmids were calculated as the proportion of barcodes for a given species with plasmid reads to the total number of barcodes assigned to that species. The average nucleotide identity (ANI) between the plasmids and the genomic sequences were calculated using fastANI.^[^ ^126^ ^]^

### Statistical Analysis—Pre‐Processing of Data

Raw sequencing FASTQ files were processed using a custom Python script (mb_barcode_and_trim.py) available on GitHub (https://github.com/AbateLab/MissonBioTools). This script performed barcode correction, adaptor trimming, and read demultiplexing. Barcode sequences were extracted using Cutadapt (v2.4),^[^ ^116^ ^]^ and sequences within a Hamming distance of 1 from a whitelist barcode were corrected. Reads were then demultiplexed using demuxbyname.sh from the BBMap package (v38.57).^[^ ^117^ ^]^

For reference‐based analysis, single‐cell reads were aligned to reference genomes using Bowtie2 (v2.3.5.1) with default settings.^[^ ^118^ ^]^ Alignment statistics, including coverage, mapped reads, and mean depth, were calculated using Samtools v1.12.^[^ ^119^, ^120^ ^]^ Barcode groups were filtered based on the percentage of alignment, or the purity score.

For TDA, single‐cell taxonomic classification was performed using Kraken2/Bracken^[^ ^42^, ^43^ ^]^ with the PlusPF or GTDB database, Kaiju with default database,^[^ ^45^ ^]^ and MetaPhlAn with default database.^[^ ^44^ ^]^ The predicted taxonomic purity of barcode groups was determined based on the abundance of the dominant taxa. Filtering was applied using purity thresholds ranging from 50% to 99%. The final taxonomic composition of barcode groups was visualized using UMAP clustering, implemented in Scanpy,^[^ ^121^ ^]^ to group barcode sequences by genus.

For rarefaction analysis, 10 000 reads were randomly sampled from each barcode group, and coverage was recalculated using Samtools.^[^ ^119^, ^120^ ^]^ The statistical distribution of strain abundance for synthetic communities was estimated using the *parseAlignment* and *estimateVBExpression* functions command in BitSeq (v1.16.0).^[^ ^62^ ^]^

### Statistical Analysis—Data Presentation

All plots were made in Python using Matplotlib, Pandas, and Seaborn. The gray shaded area in the regression plot was the 95% confidence interval (Fig. 2F). In the box plot, the box sides represent the 25th (Q1) and 75th (Q3) percentile, the line inside the box represents the median (Q2), and the whisker represent 1.5 tims the interquatile range from Q1 and Q3 (Figure S15b,c, Supporting Information).

### Statistical Analysis—Sample Size for Each Statistical Analysis

Four samples were analyzed by EASi‐seq: the ZymoBIOMICS synthetic community, an *E. lenta* 22‐strain mixture, a human gut microbiome sample, and a coastal water microbiome sample. After filtering, 1806, 5345, 1118, and 3417 barcode groups were generated from these samples, respectively.

### Statistical Analysis—Statistical Methods

Linear regression was used to compare the metagenomics and EASi‐seq reads (Figure 2F). UMAP clustering was performed using the Leiden algorithm with the default settings in Scanpy. The taxonomic abundance estimation output from Kraken2/Bracken, Kaiju, or MetaPhlAn was used directly for clustering without any transformation (Figures 2, 3, 4A, and 5A,C).

### Statistical Analysis—Software

All statistical analyses were conducted in Python using Pandas, NumPy, SciPy, and Scanpy. Bowtie2 (v2.3.5.1),^[^ ^118^ ^]^ SAMTools,^[^ ^119^, ^120^ ^]^ Seqtk toolkit, FastP,^[^ ^123^ ^]^ QUAST,^[^ ^124^ ^]^ SPAdes‐3.15.3,^[^ ^122^ ^]^ BitSeq,^[^ ^62^ ^]^ Cutadapt (v2.4),^[^ ^116^ ^]^ and BBMap(v.38.57),^[^ ^117^ ^]^ were used for sequencing read processing. Kraken2/Bracken,^[^ ^42^, ^43^ ^]^ Kaiju,^[^ ^45^ ^]^ and MetaPhlAn 3^[^ ^44^ ^]^ were used for taxonomic classification. UMAP clustering and dimensionality reduction were performed using Scanpy.^[^ ^121^ ^]^ The EASi‐seq and metagenomic contig barcode groups of the same samples were intergrated using the ingest function in the Scanpy with default setting.

### Ethical Statement

The human fecal sample was collected from a healthy adult donor under a UCSF IRB‐approved protocol (#14‐13821).

## Conflict of Interest

A.R.A., X.L., and B.D. filed patent applications related to EASi‐seq (WO2022251509A1). A.R.A. is a co‐founder and a shareholder of Mission Bio. All other authors have no competing interests.

## Author Contributions

X.L. and A.R.A. designed the research. X.L., L.X., B.D., and D.W. performed the single‐cell experiments, X.L. and B.D. analyzed the single cell data, C.N., J.E.B, and P.T.J. provided microbiome samples, X.L., C.N. and J.E.B performed metagenomic experiments and assembly. C.M. provided feedback regarding experimental design and interpretation of data, in addition to help planning of the manuscript. X.L. wrote the initial draft of the manuscript, A.R.A., C. M., C.N., J.E.B, and P.T.J revised the manuscript. All authors read, reviewed, and approved the manuscript.

## Supporting information



Supporting Information

Supporting Information

## Data Availability

The data that support the findings of this study are available in the supplementary material of this article.
